# The impact of early therapies for COVID-19 on death, hospitalization and persisting symptoms: a retrospective study

**DOI:** 10.1007/s15010-023-02028-5

**Published:** 2023-04-06

**Authors:** Paola Bertuccio, Melania Degli Antoni, Davide Minisci, Silvia Amadasi, Francesco Castelli, Anna Odone, Eugenia Quiros-Roldan

**Affiliations:** 1 Department of Public Health, Experimental and Forensic Medicine, University of Pavia, Pavia, Italy; 2 Unit of Infectious and Tropical Diseases, Department of Clinical and Experimental Sciences, ASST Spedali Civili Di Brescia and University of Brescia, Brescia, Italy

**Keywords:** COVID-19, Antivirals, Monoclonal antibodies, Early COVID-19 therapies, Long COVID, PCC

## Abstract

**Purpose:**

Oral antivirals (nirmatrelvir/ritonavir and molnupiravir), intravenous short treatment of remdesivir and anti-SARS-CoV-2 monoclonal antibodies (mAbs) have been used for early COVID-19 treatments in high risk of disease progression patients. The term long COVID has been used to refer to a range of new, returning, or ongoing symptoms after SARS-CoV-2 infection. Little is known about the impact of such therapies on long COVID.

**Methods:**

This is a retrospective observational study, including all outpatients evaluated from April 2021 to March 2022 in Brescia, Lombardy, northern Italy. Patients were stratified in three groups: (a) treated with mAbs, (b) treated with antivirals drugs and (c) controls (patients eligible for a or b who refused treatment). Data were collected at baseline and at month 1 and 3 (data on self-reported symptoms were collected using a telephone-administered questionnaire). We assessed early COVID-19 therapies effectiveness in preventing hospitalization, death at 1 or 3 months and persisting symptoms at 3 months after the onset of SARS-CoV-2 infection.

**Results:**

A total of 649 patients were included in the study, of which 242 (37.3%) were treated with mAbs, 197 (30.3%) with antiviral drugs and 210 (32.4%) were not treated. Patients most frequently reported cerebro-cardiovascular diseases (36.7%) followed by obesity (22%). Overall, 29 patients (4.5%) died or were hospitalized at 1 or 3-month follow-up. Death or hospitalization was positively associated with older ages, with a significant linear trend (OR 3.05; 95% CI 1.16–8.06, for patients aged 80 or more years compared to those aged less than 65). Data on long COVID at 3 months were available for 323 (49.8%) patients. A positive association emerged for females compared to men, with an OR of 2.14 (95% CI 1.30–3.53) for any symptoms. Conversely, inverse associations were found for treatment groups as compared to the control one, with significant estimates among patients treated with antiviral drugs for any symptoms (OR 0.43, 95% CI 0.21–0.87) and patients treated with mAbs for any neuro-behavioral symptoms (OR 0.48, 95% CI 0.25–0.92).

**Conclusions:**

We report beneficial effect of early use of anti-SARS-CoV-2 antivirals and mAbs on long COVID.

## Introduction

Severe Acute Respiratory Syndrome Coronavirus-2 (SARS-CoV-2) is a coronavirus first described in 2019, responsible for coronavirus disease 2019 (COVID-19). Since the beginning of the COVID-19 pandemic, massive efforts have been made to understand COVID-19 pathogenesis to develop targeted therapies and vaccines [1, 2].

Excess mortality and morbidity due to the COVID-19 pandemic among vulnerable people has encouraged the development of preventive and therapeutic strategies in the early stages of infection, especially as new variants of SARS-CoV-2 cause concern over vaccine efficacy [3–5]. Wide range drugs usage started with a Food and Drug Administration (FDA) Emergency Use Authorization (EUA) based on registry studies and real-life efficacy data [6–9].

Oral antivirals (nirmatrelvir/ritonavir and molnupiravir), intravenous short course of remdesivir and anti-SARS-CoV-2 monoclonal antibodies (mAbs) have been used for early COVID-19 treatments (within 5–7 days of symptoms onset) in high risk of disease progression patients [10, 11].

The virus evolution, along with development and availability of new vaccines, has substantially changed the natural history of COVID-19. A huge amount of data has been generated during the COVID-19 pandemic, with progressively accumulating knowledge on the disease. This allowed to evaluate the real efficacy of early treatments in terms of reduction of death and hospitalization. Today we are facing a new problem: a lot of patients after COVID-19 infection suffer, despite healing, from a variety of symptoms that persist after the acute syndrome. This phenomenon has been defined in different ways; here, we mention the definitions provided by the Centers for Disease Control and Prevention (CDC), as post-COVID conditions (PCC) or long COVID. The PCC refer to a wide range of new or ongoing symptoms that can last weeks, months or longer after the SARS-CoV-2 infection and that can worsen with physical or mental activity [12]. Although long COVID pathophysiology is still unclear, possible mechanisms can be immune dysregulation, endothelial dysfunction, auto-immunity, occult viral persistence and coagulation activation [13]. As reported in a systematic review of 57 studies, it has been estimated that more than half of COVID-19 patients experienced these conditions in a period of 6 months after the onset of the infection [14]. In addition, the most common symptoms reported by patients included functional mobility impairments, pulmonary abnormalities and mental health disorders [15].

Efforts in new therapies and vaccines development aimed to reduce new SARS-CoV-2 infections, and consequently, long-term sequelae but less is known about the impact of SARS-CoV-2 therapies on PCC. Several studies reported lower rates of long COVID symptoms in vaccinated patients and a recent prospective study showed a reduced prevalence of PCC in vaccinated patients, with a dose-dependent trend [16, 17].

The aim of our study is to assess early therapies effectiveness in preventing hospitalization, death and long COVID at 3 months after SARS-CoV-2 infection onset.

## Materials and methods

### Study design and study population

We conducted a retrospective observational study in the Brescia Province, located in the Lombardy Region, Northern Italy. We considered all outpatient subjects with mild-to-moderate COVID-19 evaluated from April 2021 to March 2022 at our COVID-19 outpatient clinic at Spedali Civili General Hospital in Brescia. Patients were consecutively enrolled, referred by general practitioners or other specialists with a positive SARS-CoV-2 nasopharyngeal swab.

Mild COVID-19 illness was defined by the presence of the following mild symptoms without requiring hospitalization: fever, cough, pharyngodinia, malaise, headache, myalgia, gastrointestinal symptoms (diarrhea, vomiting), loss of taste and smell, tachypnea, chills, nasal congestion without dyspnea or chest X-ray imaging abnormalities. Moderate illness was defined by the presence of clinical or radiographic evidence of lower respiratory tract infection with plasma oxygen saturation that exceed 94% [18].

Patients were included in the study (i.e., inclusion criteria) if: (i) aged > 18 years with a positive SARS-CoV-2 nasopharyngeal swab; (ii) reported mild/moderate symptoms onset within the prior 5 (for oral antivirals) or 7 (for intravenous short-course remdesivir or mAbs) days; and (iii) reported presence of at least one risk factors for COVID-19 progression: age > 65 years or older, oncological disease, hematological disease, solid organ or hematopoietic stem cell transplantation (SOT/HSCT), obesity (body mass index > 30), chronic kidney disease, cerebro-cardiovascular disease (coronary heart disease, peripheral arterial disease, congenital heart disease, pulmonary embolism, stroke, vascular dementia, cerebral haemorrhage), chronic obstructive pulmonary disease (COPD) or severe asthma, diabetes requiring medication, chronic liver disease, hemoglobinopathy, neurodegenerative diseases or other immunodeficiencies. Patients were excluded if they were hospitalized for COVID-19 pneumonia or if they had signs or symptoms of severe COVID-19 [19].

Patients were treated according to the indications provided by national guidelines, issued by the Italian Medicine Agency (Agenzia Italiana del Farmaco, AIFA) and were stratified into three groups: treated with mAbs, treated with antivirals drugs (nirmatrelvir/ritonavir, molnupiravir and short-course remdesivir) and controls (eligible patients who refused treatment).

Regarding mAbs, bamlanivimab/etesevimab and casirivimab/imdevimab were approved by AIFA on March 2021 [10] (first treatment at our clinic on 22nd April 2022), while sotrovimab was approved on 16th December 2021 [10], (first treatment on 5th January 2022). Concerning antiviral drugs, short-course remdesivir was approved by AIFA on 30th December 2021 [11] (first treatment on 5th January 2022), and molnupiravir was approved on 28th December 2021 [11] (first treatment on 10th January 2022). Finally, treatment with nirmatrelvir/ritonavir was approved on 31st January 2022 [11] (first treatment on 10th February 2022).

Data were collected at baseline and after 1 and 3 months since treatment administration as routine clinical practice in our center. Demographic characteristics (i.e., sex and age) and information about comorbidities, vaccination status and number of doses administered, and the presence of SARS-CoV-2 infection-related symptoms were individually collected at baseline.

**Fig. 1:**
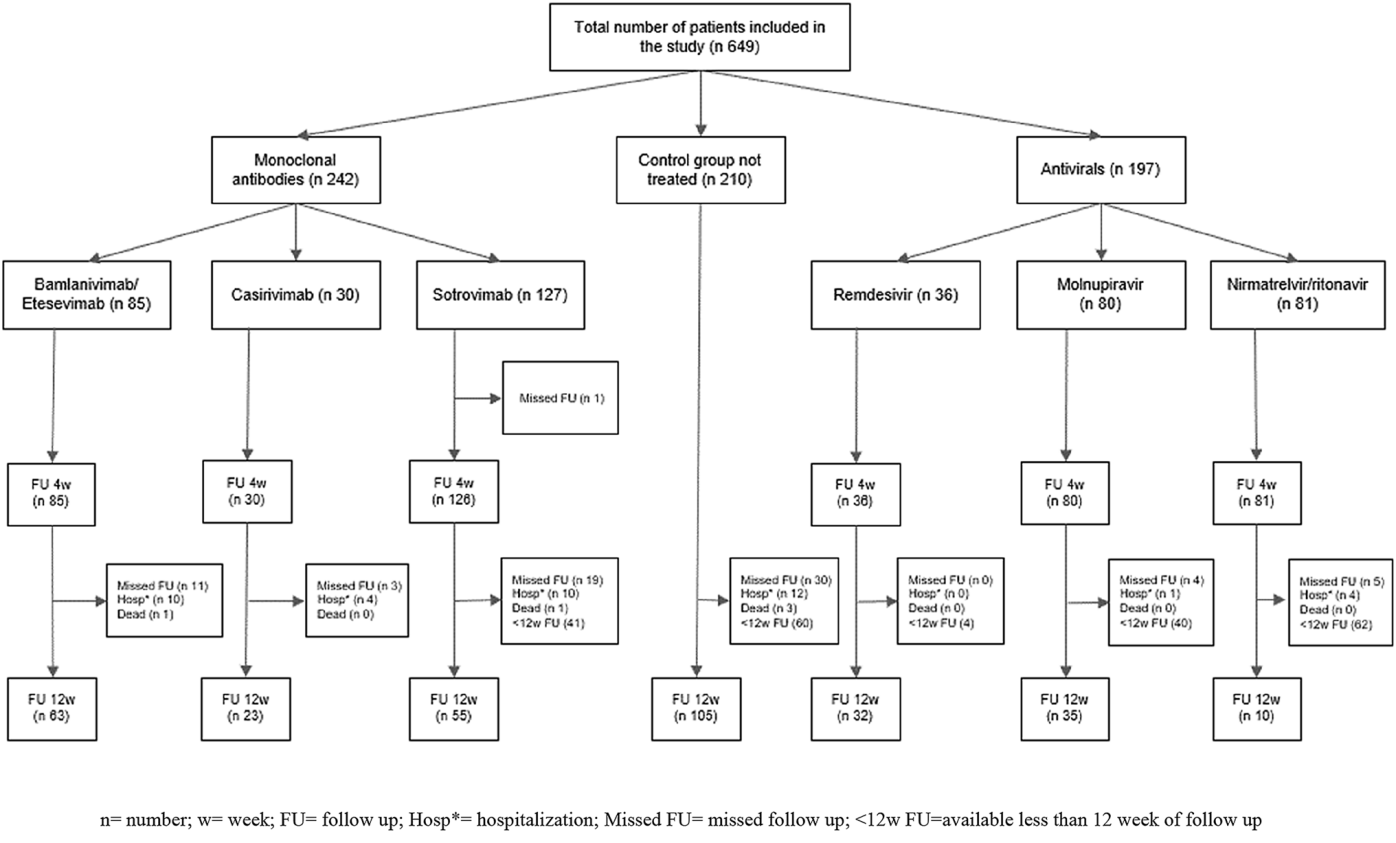
Cohort flow diagram of patients including in the study. *n* number, *w* week, *FU* follow up, Hosp* hospitalization, *Missed FU* missed follow up, *< 12w FU* available less than 12 week of follow up

At 1 and 3 months follow-up, data on self-reported symptoms were collected using a telephone-administered questionnaire. Symptoms were classified as per the U.S. CDC COVID-19 symptom list [12]. We considered long COVID symptoms as follows: general symptoms (tiredness or fatigue that interferes with daily life and fever); respiratory and heart symptoms (dyspnea, cough, pharyngodynia, chills and nasal congestion, chest pain and heart palpitations); neurological symptoms (headache, lightheadedness, muscle pain, change in smell or taste); neurobehavioral symptoms (memory and concentration deficit, sleep problems and depression or anxiety); gastrointestinal symptoms (vomiting, diarrhea). Finally, information on hospitalization or death due to COVID-19 was also registered. Follow-up at three months was available only for patients treated within 28th February 2022 (Fig. 1).

### Outcomes of interest

The primary outcome of interest was hospitalization lasting more than 24 h or death due to COVID-19, combined at 1 and 3-month follow-up. The secondary outcome was related to the long COVID defined as the persistence of at least one symptom at 3 months (considering all the symptoms as listed above), and the presence of at least one neurobehavioral symptom (including fatigue, memory and concentration deficit and anxiety/mood disorder). The latter outcome was evaluated only among the subgroup of the study outpatients for whom 3 month follow-up was available (corresponding to patients treated before 28th February 2022).

### Statistical analysis

The distribution of baseline characteristics was described as numbers and proportions in the overall sample and according to treatment group. Differences between treatment groups and selected covariates (sex, age, number of vaccine doses, presence of one or more diseases, and symptoms) were evaluated through the two-tailed Chi-square test. When the overall tests gave significant comparisons, the Bonferroni’s correction for multiple comparisons was used. Since two comparisons were made (i.e., mAbs vs. control and antiviral drugs vs. control), the significance level alpha was set as 0.025 (i.e., alpha/number of comparisons values, 0.05/2).

We estimated odds ratios (OR) of the three study outcomes and corresponding 95% confidence intervals (CI) through separate multivariate logistic regression models. Thus, each model included “death or hospitalization due to COVID-19 at 1/3 months”, “the persistence of at least one symptom at 3 months” and “the persistence of at least one neurobehavioral symptom at 3 months” as outcomes. The included covariates were sex, age (in categories < 65, 65–79, 80 + for the primary outcome and in continuous for the secondary outcomes), vaccination status (at least one dose; no doses), treatment (yes, no), treatment group (monoclonal antibodies; antiviral drugs; control group), and number of comorbidities (continuous). The models were mutually adjusted for all considered the covariates when appropriate. Tests for trend across categories were performed by including the variables as ordinal (i.e., for age groups and number of comorbidities).

During the study period, three waves of SARS-CoV-2 were described: (i) from March 2021 to May 2021 (when Alpha variant was predominant), (ii) from June 2021 to October 2021 (Delta variant dominant) and (iii) from November 2021 (Omicron variant dominant) [20]. To test the impact of SARS-CoV-2 variant dominant, a sensitive analysis was performed by stratifying the multivariable logistic regression models according to the three periods.

All the analyses were performed using the SAS software, version 9.4 (SAS Institute, Inc., Cary, NC, USA).

This study involving human subjects performed in accordance with the Helsinki Declaration of 1975 as revised in 2013. It was approved by the local ethical committee of the Spedali Civili General Hospital of Brescia (approval code NP 5363).

## Results

Overall, a total of 649 patients were included in the study. Most of the patients were infected with SARS-CoV-2 during the Omicron wave (612, 94.3%), while 23 (3.5%) patients were treated during the Alpha wave and 14 (2.2%) during the Delta wave. Among study participants, 242 (37.3%) were treated with mAbs against SARS-CoV-2, 197 (30.3%) with antiviral drugs (including nirmatrelvir/ritonavir, molnupiravir and short course of remdesivir) and 210 (32.4%) were not treated (i.e. control group) (Fig. 1). Table 1 reports study population baseline characteristics overall and by treatment group, along with the comparison between the treatment groups versus the control group. Overall, 51.6% of patients were males, with a median age of 67 years (interquartile range 54–76). Most of them were vaccinated (86%), with two or three administered doses (24% and 57.2%, respectively). Over 90% of patients presented at least one comorbidity: 51.8% had one, 28.5% two and 10% three comorbidities. Patients most frequently reported cerebro-cardiovascular diseases (36.7%) followed by obesity (22%). The most frequently reported symptoms at baseline were cough (68.4%), fever (47.1%), fatigue (36.5%), pharyngodinia (25.9%), and myalgia (23.4%). Treated patients, both with mAbs or antiviral drugs, were younger and reported more frequently comorbidities as compared to patients who did not receive treatment.

**Table 1:** Baseline demographic and clinical characteristics of the overall sample and by treatment group

	Total		Treatment group					
			Monoclonal antibodies		Antiviral drugs		Control	
	*n*	%	*n*	%	*n*	%	*n*	%
Total	649		242		197		210	
Sex	335	51.6	127	52.5	96	48.7	112	53.3
Male								
Female	314	48.4	115	47.5	101	51.3	98	46.7
Age^+^°								
18–29	19	2.9	7	2.9	6	3.0	6	2.9
30–39	35	5.4	11	4.5	7	3.6	17	8.1
40–49	59	9.1	28	11.6	19	9.6	12	5.7
50–59	117	18.0	46	19.0	48	24.4	23	11.0
60–69	128	19.7	46	19.0	36	18.3	46	21.9
70–79	179	27.6	76	31.4	43	21.8	60	28.6
80 +	110	16.9	28	11.6	37	18.8	45	21.4
Missing	2	0.3			1	0.5	1	0.5
Median (IQR)	67 (54–76)		66 (53–75)		64 (53–76)		70 (58–77)	
Number of COVID-19 vaccine doses°								
0	91	14.0	34	14.0	14	7.1	43	20.5
1	26	4.0	16	6.6	3	1.5	7	3.3
2	156	24.0	69	28.5	30	15.2	57	27.1
3	371	57.2	120	49.6	149	75.6	102	48.6
4	1	0.2	1	0.4	–	–	–	–
Missing	4	0.6	2	0.8	1	0.5	1	0.5
At least one disease^+^°								
No	45	6.9	11	4.5	4	2.0	30	14.3
Yes	604	93.1	231	95.5	193	98.0	180	85.7
Number of diseases^+^°								
1	336	51.8	108	44.6	126	64.0	102	48.6
2	185	28.5	84	34.7	45	22.8	56	26.7
3	65	10.0	30	12.4	18	9.1	17	8.1
4	17	2.6	8	3.3	4	2.0	5	2.4
5	1	0.2	1	0.4				
Risk factors for COVID-19 progression								
Oncological diseases	64	9.9	24	9.9	19	9.6	21	10.0
Hematological diseases^+^°	80	12.3	40	16.5	27	13.7	13	6.2
SOT/HSCT^+^	59	9.1	40	16.5	10	5.1	9	4.3
Obesity (body mass index > 30)	143	22.0	48	19.8	51	25.9	44	21.0
Chronic kidney diseases^+^	79	12.2	49	20.2	9	4.6	21	10.0
Cerebro-cardiovascular diseases	238	36.7	79	32.6	78	39.6	81	38.6
COPD/ asthma	105	16.2	35	14.5	33	16.8	37	17.6
Diabetes requiring medication	60	9.2	13	5.4	24	12.2	23	11.0
Chronic liver disease	25	3.9	9	3.7	7	3.6	9	4.3
Hemoglobinopathy	5	0.8	1	0.4			4	1.9
Neurodegenerative diseases	20	3.1	10	4.1	3	1.5	7	3.3
Other immunodeficiencies^+^	96	14.8	55	22.7	25	12.7	16	7.6
Treatment								
Not treated	210	32.4	-	–	–	–	210	100.0
Bamlanivimas/Etesevimab	85	13.1	85	35.1	–	–	–	–
Casirivimab/Indevimab	30	4.6	30	12.4	–	–	–	–
Sotrovimab	127	19.6	127	52.5	–	–	–	–
Remdesivir	36	5.5	–	–	36	18.3	–	–
Molnupiravir	80	12.3	–	–	80	40.6	–	–
Nirmatrelvir/Ritonavir	81	12.5	–	–	81	41.1	–	–
At least one symptom at first contact^+^°	16	2.5	1	0.4	–	–	15	7.1
No^□^								
Yes	633	97.5	241	99.6	197	100.0	195	92.9
Number of symptoms at diagnosis^+^								
1	140	21.6	43	17.8	48	24.4	49	23.3
2	224	34.5	89	36.8	64	32.5	71	33.8
3	148	22.8	55	22.7	49	24.9	44	21.0
4	70	10.8	29	12.0	21	10.7	20	9.5
5	34	5.2	17	7.0	12	6.1	5	2.4
6	8	1.2	3	1.2	2	1.0	3	1.4
7	8	1.2	5	2.1	–	–	3	1.4
9	1	0.2	–	–	1	0.5	–	–
Symptoms at diagnosis								
Fever^+^	306	47.1	139	57.4	72	36.5	95	45.2
Cough°	444	68.4	169	69.8	145	73.6	130	61.9
Pharyngodinia^+^°	168	25.9	65	26.9	66	33.5	37	17.6
Nasal congestion°	45	6.9	6	2.5	29	14.7	10	4.8
Dyspnea	46	7.1	13	5.4	16	8.1	17	8.1
Ageusia/Dysgeusia	36	5.5	19	7.9	7	3.6	10	4.8
Anosmia	23	3.5	12	5.0	3	1.5	8	3.8
Fatigue	237	36.5	97	40.1	65	33.0	75	35.7
Headache	86	13.3	36	14.9	27	13.7	23	11.0
Myalgia	152	23.4	62	25.6	45	22.8	45	21.4
Chills	7	1.1	2	0.8	3	1.5	2	1.0
Gastrointestinal symptoms	45	6.9	20	8.3	10	5.1	15	7.1

*SOT/HSCT* Solid Organ Transplantation/Hematopoietic Stem-Cell Transplantation, *COPD* Chronic Obstructive Pulmonary Disease

^+^*p* value for chi-square test < 0.025 (after Bonferroni’s correction for multiple comparison 0.05/2) means that the comparison between monoclonal antibodies vs. control group resulted statistically significant

°*p* value for chi-square test < 0.025 (after Bonferroni’s correction for multiple comparison 0.05/2) means that the comparison between antiviral drugs vs. control group resulted statistically significant

^□^Patients reached hospital when COVID-19 was asymptomatic after a symptomatic period

Numbers and percentages of “death or hospitalization due to COVID-19 at 1/3 months” according to selected covariates and results from the multivariable logistic regression models are reported in Table 2. Overall, 29 patients (4.5%) died or were hospitalized. Death or hospitalization was positively associated with older ages. As compared to patients aged less than 65 years, those aged 80 years or more showed an OR of 3.05 (95% CI 1.16–8.06) with a significant linear trend (*p* for trend: 0.033). No statistically significant associations emerged for the other factors considered including sex, vaccination status, treatment group or comorbidities.

**Table 2:** Numbers and percentages of “death or hospitalisation for COVID-19 at 1/3 months” according to selected covariates and results from the logistic regression models

	Death or hospitalisation for COVID-19 at 1/3 months				
	No		Yes		Adjusted OR^a^ (95% CI)
	*n*	%	*n*	%	
Total	620	95.5	29	4.5	
Sex					
Female	301	95.9	13	4.1	1^b^
Male	319	95.2	16	4.8	1.21 (0.56–2.59)
Age group					
< 65	275	96.8	9	3.2	1^b^
65–79	243	96.0	10	4.0	1.21 (0.48–3.07)
≥ 80	100	90.9	10	9.1	**3.05 (1.16–8.06)**
Missing	2	–	–	–	
Chi-square for trend					0.033
Vaccination					
No	85	93.4	6	6.6	1^b^
Yes	531	95.8	23	4.2	0.50 (0.19–1.32)
Missing	4	–	–	–	–
Treatment					
No	199	94.8	11	5.2	1^b^
Yes	421	95.9	18	4.1	0.87 (0.39–1.92)
Treatment group					
Control	199	94.8	11	5.2	1
Monoclonal	224	92.6	18	7.4	1.61 (0.72–3.61)
Antiviral	197	100.0	0	0.0	–
At least one disease					
No	43	95.6	2	4.4	1^b^
Yes	577	95.5	27	4.5	1.10 (0.24–4.99)
Comorbidity					
No diseases	43	95.6	2	4.4	1^b^
One disease	325	96.7	11	3.3	0.83 (0.17–4.02)
Two or more diseases	252	94.0	16	6.0	1.44 (0.31–6.82)
Chi-square for trend					0.060

The estimates resulted statistically significant were highlighted in bold

^a^Estimates obtained through logistic regression models mutually adjusted by sex, age group (< 65, 65–79, 80 +), vaccination (yes, no), treatment (yes, no), and number of diseases (continuous), when appropriate

^b^Reference category

**Fig. 2:**
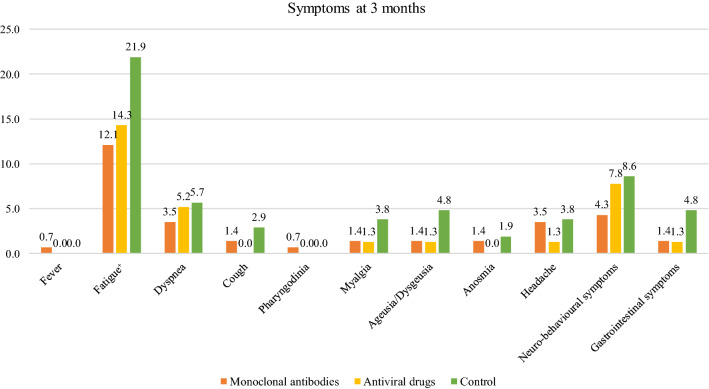
Percentages of symptoms reported at 3 months according to the treatment group. ^+^*p*-value for chi-square test equal to 0.038 for the comparison between monoclonal antibodies vs. control group for fatigue (i.e., borderline statistically significant since a *p*-value < 0.025 was considered statistically significant, after the Bonferroni’s correction)

Data on COVID-19 symptoms at 3 months were available for 323 (49.8%) patients. Table 3 reports baseline characteristics overall and by treatment group, along with comparison between the treatment groups versus the control group. Similar distributions for demographic characteristics, vaccination status and comorbidities were observed. Regarding symptoms, fatigue was the most frequently reported, followed by the neuro-behavioral symptoms. All symptoms were more prevalent in the control group compared to mAbs or antiviral treated patients, though only the fatigue symptom showed a borderline statistically significant difference (*p* value for Chi-square test: 0.038) between the group of patients treated with mAbs vs. the control group (Fig. 2).

**Table 3:** Baseline demographic and clinical characteristics of the subgroup of 323 patients evaluated for the “long COVID” outcome, overall and by treatment group

	Total, *N* = 323		Treatment group					
			Monoclonal antibodies, *N* = 141		Antiviral drugs, *N* = 77		Control, *N* = 105	
	*n*	%	*n*	%	*n*	%	*n*	%
Sex°								
Male	174	53.9	79	56.0	32	41.6	63	60.0
Female	149	46.1	62	44.0	45	58.4	42	40.0
Age°								
18–29	12	3.7	5	3.5	2	2.6	5	4.8
30–39	12	3.7	4	2.8	1	1.3	7	6.7
40–49	33	10.2	16	11.3	9	11.7	8	7.6
50–59	62	19.2	24	17.0	24	31.2	14	13.3
60–69	60	18.6	30	21.3	11	14.3	19	18.1
70–79	95	29.4	49	34.8	11	14.3	35	33.3
80 +	47	14.6	13	9.2	18	23.4	16	15.2
Missing	2	0.6			1	1.3	1	1.0
Median (IQR)	66 (54–75)		67 (55–75)		60.5 (53–76.5)		69 (56.5–76)	
Number of COVID-19 vaccine doses°								
0	50	15.5	19	13.5	8	10.4	23	21.9
1	16	5.0	12	8.5	1	1.3	3	2.9
2	111	34.4	55	39.0	19	24.7	37	35.2
3	145	44.9	55	39.0	49	63.6	41	39.0
Missing	1	0.3	–	–	–	–	1	1.0
At least one disease^+^°								
No	20	6.2	6	4.3	–	–	14	13.3
Yes	303	93.8	135	95.7	77	100.0	91	86.7
Number of diseases°								
1	164	50.8	70	49.6	45	58.4	49	46.7
2	101	31.3	45	31.9	24	31.2	32	30.5
3	23	7.1	13	9.2	5	6.5	5	4.8
4	15	4.6	7	5.0	3	3.9	5	4.8
Risk factors for COVID-19 progression								
Oncological diseases	28	8.7	10	7.1	5	6.5	13	12.4
Hematological diseases	35	10.8	19	13.5	10	13.0	6	5.7
SOT/HSCT^+^	35	10.8	23	16.3	7	9.1	5	4.8
Obesity (body mass index > 30)	77	23.8	27	19.1	26	33.8	24	22.9
Chronic kidney diseases	40	12.4	26	18.4	5	6.5	9	8.6
Cerebro-cardiovascular diseases	109	33.7	48	34.0	27	35.1	34	32.4
COPD/moderate-severe asthma	56	17.3	21	14.9	15	19.5	20	19.0
Diabetes requiring medication^+^	35	10.8	9	6.4	10	13.0	16	15.2
Chronic liver disease	15	4.6	5	3.5	3	3.9	7	6.7
Hemoglobinopathy	3	0.9					3	2.9
Neurodegenerative diseases	6	1.9	3	2.1			3	2.9
Other immunodeficiencies^+^	56	17.3	36	25.5	12	15.6	8	7.6
Treatment								
Not treated	105	32.5	–	–	–	–	105	100.0
Bamlanivimab/Etesevimab	63	19.5	63	44.7	–	–	–	–
Casirivimab/Indevimab	23	7.1	23	16.3	–	–	–	–
Sotrovimab	55	17.0	55	39.0	–	–	–	–
Remdesivir	32	9.9	–	–	32	41.6	–	–
Molnupiravir	35	10.8	–	–	35	45.5	–	–
Nirmatrelvir/Ritonavir	10	3.1	–	–	10	13.0	–	–

*SOT/HSCT* Solid Organ Transplantation/Hematopoietic Stem-Cell Transplantation, *COPD* Chronic Obstructive Pulmonary Disease

^+^*p* value for chi-square test < 0.025 (after Bonferroni’s correction for multiple comparison 0.05/2) means that the comparison between monoclonal antibodies vs. control group resulted statistically significant

°*p* value for chi-square test < 0.025 (after Bonferroni’s correction for multiple comparison 0.05/2) means that the comparison between antiviral drugs vs. control group resulted statistically significant

Distribution of the two secondary outcomes related to long COVID according to selected covariates and results from the multivariable logistic regression models are reported in Table 4. Prevalence of long COVID (defined as the presence of at least one symptom at 3 months) was 29.1%. Females showed a positive association with long COVID, with an OR of 2.14 (95% CI 1.30–3.53) as compared to men. Patients treated with antiviral drugs showed an inverse association with long COVID (OR 0.43, 95% CI 0.21–0.87 as compared to not treated patients). According to the definition of long COVID, the presence of at least one neuro-behavioural symptom was an outcome. Prevalence was 19.5% with a similar pattern of associations. Females reported more frequently a persistence of neurobehavioral symptoms than men (OR 2.69, 95% CI 1.50–4.83). Patients who were treated with mAbs showed an OR of 0.48 (95% CI 0.25–0.92) as compared to those in the control group.

**Table 4:** Numbers and percentages of “long COVID” according to selected covariates and results from the logistic regression models

	At least one symptom					At least one neuro-behavioural symptom				
	No		Yes		Adjusted OR^a^ (95% CI)	No		Yes		Adjusted OR^a^ (95% CI)
	*n*	%	*n*	%		*n*	%	*n*	%	
Total	229	70.9	94	29.1		260	80.5	63	19.5	
Sex										
Male	135	77.6	39	22.4	1^b^	151	86.8	23	13.2	1^b^
Female	94	63.1	55	36.9	**2.14 (1.30–3.53)**	109	73.2	40	26.8	**2.69 (1.50–4.83)**
Age group										
≤ 65	98	67.1	48	32.9	1^b^	116	79.5	30	20.5	1^b^
> 65	131	74.0	46	26.0	0.68 (0.42–1.12)	144	81.4	33	18.6	0.83 (0.47–1.47)
Vaccination										
No	34	68.0	16	32.0	1^b^	40	80.0	10	20.0	1^b^
Yes	194	71.3	78	28.7	1.06 (0.53–2.10)	219	80.5	53	19.5	1.09 (0.50–2.40)
Treatment										
No	69	65.7	36	34.3	1^b^	78	74.3	27	25.7	1^b^
Yes	160	73.4	58	26.6	0.64 (0.38–1.09)	182	83.5	36	16.5	**0.49 (0.27–0.89)**
Treatment group										
Control	69	65.7	36	34.3	1^b^	78	74.3	27	25.7	1^2^
Monoclonal antibodies	99	70.2	42	29.8	0.78 (0.44–1.36)	119	84.4	22	15.6	**0.48 (0.25–0.92)**
Antiviral drugs	61	79.2	16	20.8	**0.43 (0.21–0.87)**	63	81.8	14	18.2	0.51 (0.24–1.10)

The estimates resulted statistically significant were highlighted in bold

^a^Estimates obtained through logistic regression models mutually adjusted by sex, age (continuous), vaccination (yes, no), treatment (yes, no), and number of diseases (continuous), when appropriate

^b^Reference category

Results from the sensitive analysis did not show any differences between the three periods, where prevalent variants were respectively Alpha, Delta and Omicron (data not shown).

## Discussion

Currently, people are continuing to get infected despite being vaccinated for SARS-CoV-2, but emergency use authorized COVID-19 treatments are available today and, if they started early, they would show to be effective for people with mild/moderate COVID-19 even though other health conditions or diseases.

These treatments demonstrated their efficacy during the Delta variant wave and focused on unvaccinated people [6–9]; currently they have been also used in frail vaccinated patients infected with Omicron SARS-CoV-2 variant but few real-life data regarding their benefit are available. Here, we describe real-life efficacy of early treatments for mild/moderate COVID-19 (mAbs or antivirals) comparing outcomes (hospitalization and death rates) with a group of not treated subjects (they refused treatment despite clinical indication). Moreover, relationship between treatment and persistence of symptoms (self-reported) at 3 months onset after treatment has been evaluated.

Other experimental early treatments are investigating (e.g., convalescent plasma) but we focused on therapies approved by AIFA to include a large number of patients [21–23].

Findings from our retrospective study conducted in Northern Italy over the period April 2021–March 2022 among frail outpatients patients demonstrate that death or hospitalization at 1 or 3 months from the infection was more frequent among older patients (with a significant linear trend), being three times higher among patients older than 80 than those aged less than 65 years (OR 3.05 95%CI 1.16–8.08), independently from sex, early treatment administration, vaccination status or number of comorbidities [24]. Regarding long COVID outcomes, around 29.1% patients reported at least one symptom after 3 months and fatigue was the most frequent complaint (15.5%) followed by neurobehavioral symptoms (6%). A protective effect of early use of antivirals (0.43; 95% CI 0.21–0.87 as compared to the control group) emerged for long COVID (any symptoms), as well as for any neuro-behavioral symptoms (0.48; 95% CI 0.25–0.92).

The Lombardy Region was the first and most heavily by the pandemic in Europe [25]. On November–December 2021, we faced with the fourth wave of SARS-CoV-2 epidemic caused mainly by the Omicron variant, with the larger number of infections (despite a rate of vaccination around 90%) and lower rate of deaths from the beginning of pandemic [26]. The present study was performed when Omicron variant started to be predominant [27]. While Omicron variant, due to the multiple changes across its genome, partially evades/escapes natural or vaccine-induced immunity, infection with this variant resulted with a reduced clinical severity [28]. Therefore, although viral genome analysis was not available, we could assume that the low rate of hospitalization/death without differences between groups, except for very elderly patients, could be attributed to Omicron, the main circulating variant during the study period [29].

Early during pandemic, it was observed that several symptoms persisted for a considerable time after the acute phase of COVID-19 (generally 4 weeks after diagnosis). These conditions were defined as long COVID or PCC and the lack of standard methods for diagnosis until now causes an extreme heterogeneity in clinical manifestations and prevalence [30]. Factors such as age, comorbidities, need of mechanical ventilation or intensive care support during acute disease were controversially associated with PCC [31–34]. It may depend on heterogeneity of the cohorts, severity of symptoms collected and time of follow-up [31–34].

More intriguing are the possible mechanisms underlying this syndrome that seems to be complex, probably multifactorial and not yet elucidated [35]. Some authors argue that the persistence of SARS-CoV-2 or their antigens can induce a lasting immune response including inflammation, autoimmunity, and disruption of the microbiome [36] and all COVID-19 severities seem to have a similar risk of developing long COVID [37].

In a recent meta-analysis, the global estimated prevalence of long COVID in non-hospitalized patients was 34% (95% CI 0.25, 0.46) [38] with a higher risk among women than men [39] as also confirmed in our study.

Several studies have described a protective association between SARS-CoV-2 vaccination and long COVID symptoms [17, 40] and this protection seems to persist also in breakthrough infections [41]. We have not found any effect of vaccination about the risk of long COVID at 3 months onset in any of the adjusted logistic models (any symptoms, neurobehavioral symptoms). Most of risk factors for COVID-19 clinical progression (considered as criteria of inclusion in our study) might linked to immune deficiency and to vaccine low response.

The different effects on the risk of symptoms persistence at 3 months onset between mAbs and antivirals found in our study is unknown.

SOLIDARITY trial did not demonstrate long-term benefits (1 year of follow-up) of remdesivir in patients who had hospitalized due to COVID-19 [42]. We have not studied antivirals separately because subgroups were too small to give reliable information but evaluated all together they seem to protect from PCC (any symptom) at 3 months.

One of the hypotheses regarding pathogenesis of long COVID is that it might be linked to prolonged inflammation probably due to viral antigens persistence [43]. Monoclonal antibodies prevent the viral attachment to the cells blocking the binding of the virus to the human angiotensin-converter enzyme 2 receptor (ACE2) that is highly expressed on the surface of oligodendrocytes, which could explain the virus proliferation in nerve tissue cells and subsequent neurological damage and sequelae [44, 45]. Our data suggest a protective effect of mAbs on neurobehavioral symptoms including fatigue that was the most prevalent symptom. On the other hand, antivirals inhibit viral replication, but lingering virus and viral antigens are not blocked and they could prolong inflammation.

Our study has two main limitations. First, its retrospective study design, second, the small sample size of the subgroups. Moreover, symptoms are self-reported and graded as binary (presence/absence) and intensity was not considered; finally, we do not have information regarding potential symptomatic treatments. However, as no specific treatment has been proved effective for long COVID, this may likely not influence our results.

In conclusion, we found some beneficial effect of mild/moderate COVID-19 treatment on the persistence of symptoms at 3 months after acute disease. Different underlying mechanisms might be responsible for different PCC symptoms and in that case, treatments should have different effects.

## Data Availability

All de-identified individual participant data collected during the study, in addition to the study protocol, will be made available immediately following the publication of this article to anyone who wishes to access the data.
